# Synthesis and crystal structure of 1,3-bis­(acet­oxymeth­yl)-5-{[(4,6-di­methyl­pyridin-2-yl)amino]­methyl}-2,4,6-tri­ethyl­benzene

**DOI:** 10.1107/S2056989024007515

**Published:** 2024-08-13

**Authors:** Manuel Stapf, Venugopal Rao Miyyapuram, Wilhelm Seichter, Monika Mazik

**Affiliations:** a Institut für Organische Chemie, Technische Universität Bergakademie Freiberg, Leipziger Str. 29, 09596 Freiberg/Sachsen, Germany; b Clinical Research Products Management Center (CRPMC) Bioservices Thermo Fisher Scientific, 1055 First Street Rockville/Maryland 20850 USA; Universidade de Sâo Paulo, Brazil

**Keywords:** crystal structure, tripodal mol­ecule, hydrogen bonding, C—H⋯π inter­actions

## Abstract

In the crystal structure of the title compound, C_26_H_36_N_2_O_4_, the tripodal mol­ecule exists in a conformation in which the substituents attached to the central arene ring are arranged in an alternating order above and below the ring plane. The heterocyclic unit is inclined at an angle of 79.6 (1)° with respect to the plane of the benzene ring. In the crystal, the mol­ecules are connected *via* N—H⋯O bonds, forming infinite supra­molecular strands. Inter­strand association involves weak C—H⋯O and C—H⋯π inter­actions, with the pyridine ring acting as an acceptor in the latter case.

## Abstract 2

In the crystal structure of the title compound, C_26_H_36_N_2_O_4_, the tripodal mol­ecule adopts a conformation in which the substituents attached to the central benzene ring are arranged in an alternating order above and below the ring plane.

## 1. Chemical context

Recognition units based on 2-amino­pyridine have proved to be valuable building blocks for the construction of artificial carbohydrate receptors that act *via* non-covalent inter­actions (Mazik *et al.*, 2004 ▸, 2005 ▸; Mazik, 2009 ▸, 2012 ▸; Lippe & Mazik, 2015 ▸; Seidel *et al.*, 2023 ▸). Such units are able to participate in the formation of hydrogen-bonding motifs similar to those observed in natural complexes for the primary amide groups (side chains of asparagine and glutamine). The latter are used by carbohydrate-binding proteins in combination with other functional groups such as hy­droxy, carb­oxy, imidazolyl and isopropyl groups (side chains of serine, aspartic acid, histidine and valine, respectively). The use of a combination of different functional groups enables not only the formation of neutral and charge-reinforced hydrogen bonds, but also of C—H⋯π inter­actions and numerous van der Waals contacts, and is responsible for the observed binding selectivities and efficiencies of the proteins (Quiocho, 1989 ▸; Sharon & Lis, 2007 ▸; Gabius, 2009 ▸; Gabius *et al.*, 2011 ▸). Our studies with various acyclic and macrocyclic artificial receptors have also shown that selective and effective binding is favourably influenced by the involvement of different functional groups in the binding process. Among the acyclic receptor mol­ecules, 1,3,5-substituted 2,4,6-tri­alkyl­benzene derivatives have been studied particularly intensively (Lippe *et al.*, 2015 ▸; Kaiser *et al.*, 2019 ▸; Stapf *et al.*, 2020*a* ▸; Köhler *et al.*, 2020 ▸, 2021 ▸, 2024 ▸), and different binding properties have been observed depending on the nature of the receptor building blocks. In this article, we describe the crystal structure of 1,3-bis­(acet­oxy­meth­yl)-5-{[(4,6-di­methyl­pyridin-2-yl)amino]­meth­yl}-2,4,6-tri­ethyl­benzene, which is a precursor for the synthesis of a tri­ethyl­benzene derivative bearing a 2-amino­pyridine-based recognition moiety and two hy­droxy­methyl groups.
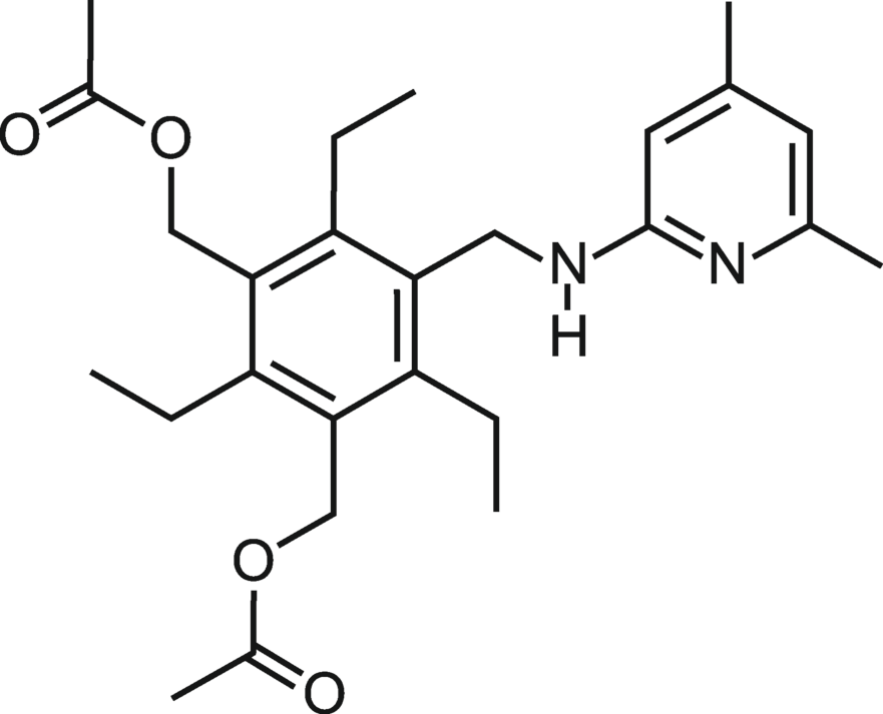


## 2. Structural commentary

**Figure 1:**
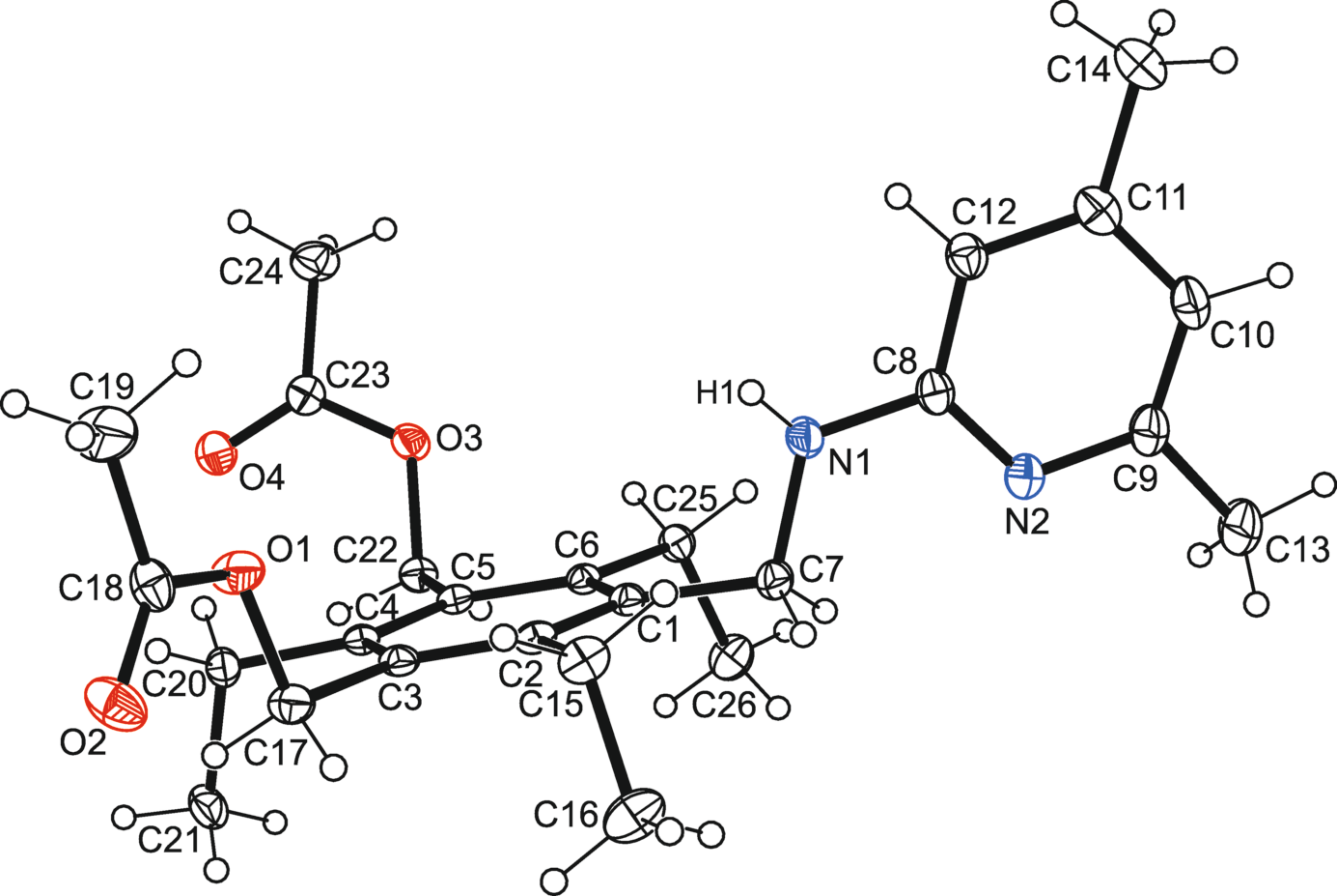
Perspective view of the title mol­ecule including atom labelling. Anisotropic displacement ellipsoids are drawn at the 50% probability level.

The crystal structure of the title compound, C_26_H_36_N_2_O_4_, was solved in the monoclinic space group *P*2_1_/*n* with the asymmetric unit containing one mol­ecule. As shown in Fig. 1 ▸, the mol­ecule adopts a conformation in which the pyridinyl­amino moiety and the two acet­oxy groups are located on one side of the central benzene ring, whereas the ethyl substituents are directed to the opposite side of the ring plane (*ababab* arrangement; Das & Barbour, 2008*a* ▸,*b* ▸, 2009 ▸; Koch *et al.*, 2017 ▸; Schulze *et al.*, 2017 ▸). The mol­ecule exists in a strongly distorted conformation with torsion angles of −166.6 (1) (*anti*) and −121.3 (1)° (eclipsed) along the C_ar­yl_—C—O—C sequences and an inter­planar angle of 79.6 (1)° between the aromatic rings. The conformation appears to be stabilized by an intra­molecular C—H⋯N hydrogen bond [*d*(H⋯N) = 2.65 Å] and a C—H⋯O bond [*d*(H⋯O) = 2.52 Å] involving the ethyl hydrogen atoms H25*A*, H25*B* and the acceptor positions N1 and O3 (Table 1 ▸).

## 3. Supra­molecular features

**Figure 2:**
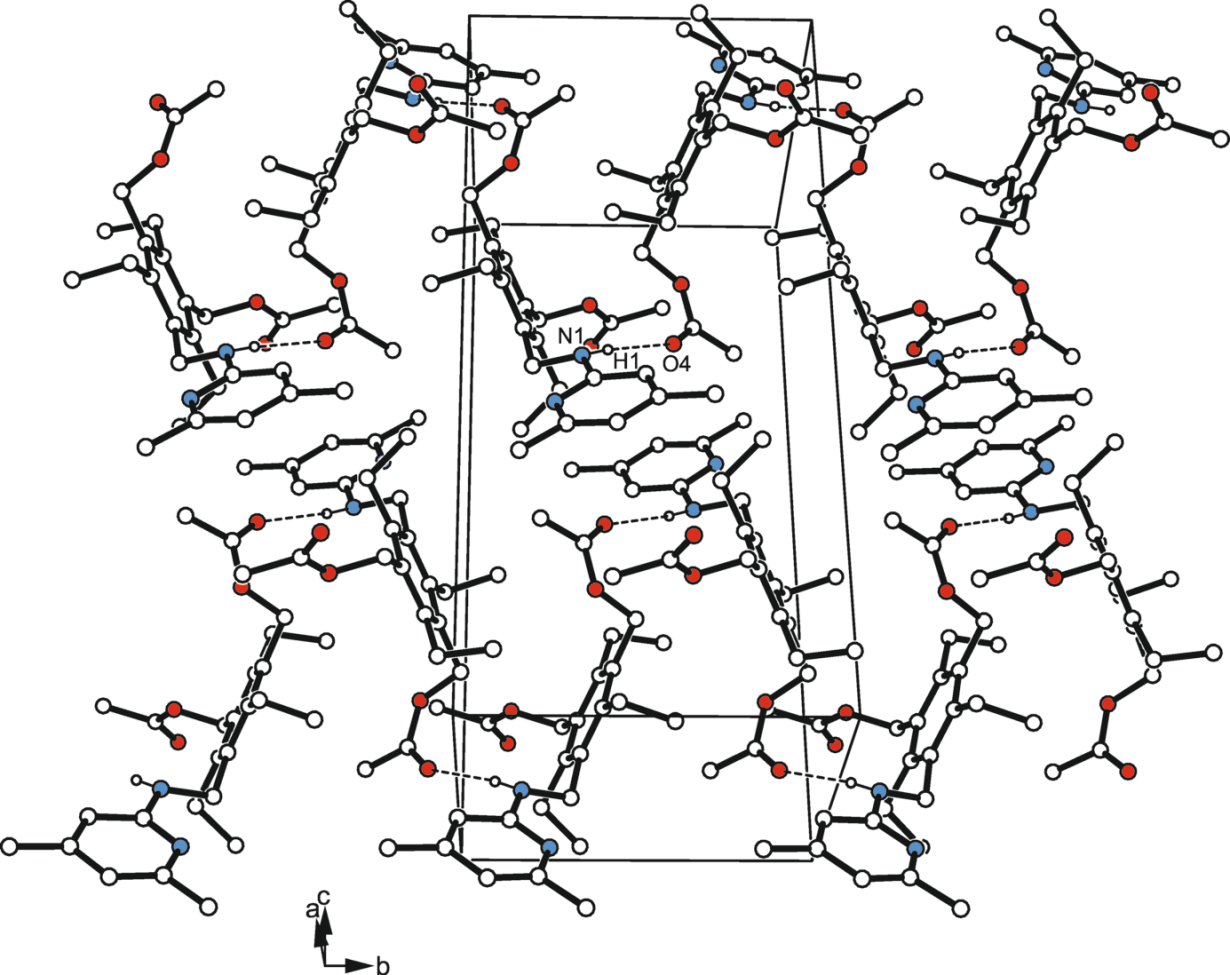
Packing diagram of the title compound. The N—H⋯O hydrogen bonds are shown as dashed lines.

The crystal structure is composed of zigzag-like strands of N-H⋯O=C bonded mol­ecules [N1—H1⋯O4, 2.10 (1) Å, 173 (1)°], that extend parallel to the crystallographic *b* axis (Fig. 2 ▸). Inter­strand association is confined to only one C—H⋯π contact (Nishio *et al.*, 2009 ▸, 2012 ▸) per mol­ecule with the pyridine ring acting as an acceptor [C14—H14*B*⋯*Cg*, 2.73 Å, 147°] as well as a weak C—H⋯O bond (Desiraju & Steiner, 1999 ▸) involving the oxygen atom O2 [C19—H19*C*⋯O2, 2.61 Å, 117°].

## 4. Database survey

Our previous studies have shown that representatives of 1,3,5-substituted 2,4,6-tri­alkyl­benzenes with side arms bearing different functional groups have a better ability to discriminate between various carbohydrate substrates than compounds possessing identical functionalized side arms. In this context, the combination of 2-amino­pyridine-based building blocks with other functional groups was shown to provide compounds capable of acting as effective and selective carbohydrate receptors. The search in the Cambridge Structural Database (CSD, Version 5.45, update June 2024; Groom *et al.*, 2016 ▸) for such mol­ecules with one or two pyridin-2-yl-amino­methyl unit(s) yielded thirteen hits. All crystal structures of the tri­ethyl­benzene derivatives listed below have in common that the tripodal mol­ecules adopt a conformation with an alternating arrangement of the substituents above and below the plane of the central benzene ring. The crystal structures of the monohydrate and the methanol solvate of {1-[(3,5-bis­{[(4,6-di­methyl­pyridin-2-yl)amino]­meth­yl}-2,4,6-tri­ethyl­benz­yl)amino]­cyclo­pent­yl}methanol (CADTAG, CADTEK; Stapf *et al.*, 2020*b* ▸) as well as that of the methanol solvate of 1-{[*N*,*N*′-bis­(*tert*-but­oxy­carbon­yl)guanidino]meth­yl}-3,5-bis­{[(6-methyl­pyridin-2-yl)amino]­meth­yl}-2,4,6-tri­ethyl­benzene (HEXVAI; Mazik & Cavga, 2007 ▸) are composed of inversion-symmetric mol­ecular dimers in which the water or methanol mol­ecules are enclosed. Thus, the dimers are held together by solvent-mediated hydrogen bonds. In a similar way, the solvent-free crystal structures of 1,3-bis­{[*N*,*N*-bis­(2-hy­droxy­eth­yl)amino]­meth­yl}-5-{[(4,6-di­methyl­pyridin-2-yl)amino]­meth­yl}-2,4,6-tri­ethyl­benzene (BEFGAY; Stapf *et al.*, 2022 ▸) and 1-{[*N*,*N*-bis­(eth­oxy­carbonyl­meth­yl)amino]­meth­yl}-3,5-bis­{[(6-methyl­pyridin-2-yl)amino]­meth­yl}-2,4,6-tri­methyl­benz­ene (KEGWID; Mazik & Cavga, 2006 ▸) also consist of centrosymmetric dimers as the smallest supra­molecular entity. In the crystal structure of 1,3-bis­{[*N*-(1,10-phenanthrolin-2-ylcarbon­yl)amino]­methyl-5-{[(4,6-di­methyl­pyridin-2-yl)amino]­meth­yl}-2,4,6-tri­ethyl­benzene (TUGVEX; Mazik *et al.*, 2009 ▸), two water mol­ecules and one ethanol mol­ecule are accommodated in the binding pocket created by the heterocyclic units (one pyridinyl and two phenanthrolinyl groups) of the host mol­ecule. The related compound 1-{[*N*-(1,10-phen­an­throlin-2-ylcarbon­yl)amino]­meth­yl}-3,5-bis­{[(4,6-di­methyl­pyridin-2-yl)amino]­meth­yl}-2,4,6-tri­ethyl­benzene (ROKJEH, ROKJEH01; Mazik & Hartmann, 2008 ▸; Mazik *et al.*, 2009 ▸), possessing one phenanthrolinyl and two pyridinyl groups, encloses three water mol­ecules in the binding pocket. Both host–water/ethanol aggregates are stabilized by O—H⋯O, N—H⋯O and O—H⋯N hydrogen bonds. In the crystal structures of the formamide monosolvate and the *n*-propanol/H_2_O solvate of 1-{[2,6-bis­(hy­droxy­meth­yl)-4-methyl­phen­oxy]meth­yl}-3,5-bis­{[(4,6-di­methyl­pyridin-2-yl)amino]­meth­yl}-2,4,6-tri­ethyl­benzene (FIZDOL, FIZDUR; Stapf *et al.*, 2023 ▸), the tripodal host mol­ecules adopt similar conformations despite the different solvent mol­ecules. 1-(Bromo­meth­yl)-3,5-bis­{[(4,6-di­methyl­pyridin-2-yl)amino]­meth­yl}-2,4,6-tri­ethyl­benzene was found to crystallize as a diethyl ether solvate (BIYTOT; Mazik & Kuschel, 2008 ▸), with the ether oxygen bound to one of the amino groups by hydrogen bonding. Finally, the crystal structures of triethylbenzene derivatives bearing one or two cationic moieties, namely 3-methylpyridinium group(s), in combination with 4,6-dimethylpyridin-2-yl unit(s) (hexafluorophosphate salts; ZITRAZ and ZITRON, respectively; Weisse *et al.*, 2023 ▸) should be mentioned.

## 5. Synthesis and crystallization

A suspension of 1,3-bis­(bromo­meth­yl)-5-{[(4,6-di­methyl­pyridin-2-yl)amino]­meth­yl}-2,4,6-tri­ethyl­benzene (0.30 g, 0.62 mmol) and anhydrous sodium acetate (0.42 g, 5.12 mmol) in acetic acid (3 mL) was stirred at 373 K for 12 h. The solvent was evaporated under reduced pressure. To the obtained white solid, water (3 mL) and CH_2_Cl_2_ (3 mL) were added. The aqueous phase was extracted twice with CH_2_Cl_2_ (5 mL). The combined organic extracts were treated with a saturated NaHCO_3_ solution (3 mL), washed with water (5 mL), dried (Na_2_SO_4_) and then concentrated. The pale yellow resin was recrystallized from methanol to give the title compound (0.17 g, 62%) as colourless crystals. Single crystals suitable for X-ray diffraction were obtained by slow evaporation from a solution of the title compound in *N*,*N*-di­methyl­acetamide.

*Analytical data*: m.p. 429–431 K; ^1^H NMR (500 MHz, CDCl_3_, ppm): *δ* = 1.18–1.22 (*m*, 9H, CH_3_), 2.09 (*s*, 6H, CH_3_), 2.24 (*s*, 3H, CH_3_), 2.36 (*s*, 3H, CH_3_), 2.76 (*q*, 6H, *J* = 7.6 Hz, CH_2_), 4.15 (*br s*, 1H, NH), 4.39 (*d*, 2H, *J* = 4.2 Hz, CH_2_), 5.21 (*s*, 4H, OCH_2_), 6.08 (*s*, 1H, ar­yl), 6.36 (*s*, 1H, ar­yl); ^13^C NMR (125 MHz, CDCl_3_, ppm): *δ* = 16.3, 16.5 (CH_3_), 21.0 (CH_3_), 21.1 (CH_3_), 22.8, 23.0 (CH_2_), 24.1 (CH_3_), 40.4 (NHCH_2_), 60.9 (OCH_2_), 103.5, 114.0, 130.0, 133.2, 145.4, 145.8, 148.9, 156.7, 158.1 (all ar­yl), 171.1 (C=O); MS (APCI): *m*/*z* calculated for C_26_H_37_N_2_O_4_: 441.3 [*M* + H]^+^, found 441.2. Elemental analysis for C_26_H_36_N_2_O_4_ (%): calculated C 70.88, H 8.24, N 6.36; found C 70.68, H 8.20, N 6.40. TLC: R_*f*_ = 0.41 [SiO_2_, toluene/ethyl acetate 3:1 (*v*/*v*)].

The educt, 1,3-bis­(bromo­meth­yl)-5-{[(4,6-di­methyl­pyridin-2-yl)amino]­meth­yl}-2,4,6-tri­ethyl­benzene, was synthesized according to the reported procedure (Weisse *et al.*, 2023 ▸).

## 6. Refinement

Crystal data, data collection and structure refinement details are summarized in Table 2 ▸. The non-hydrogen atoms were refined anisotropically. All hydrogen atoms were positioned geometrically and refined isotropically using a riding model with C—H = 0.98–0.99 Å (alk­yl), 0.95 Å (ar­yl); *U*_iso_(H) = 1.2–1.5*U*_eq_(C).

## Supplementary Material

Crystal structure: contains datablock(s) I. DOI: 10.1107/S2056989024007515/ex2085sup1.cif

Structure factors: contains datablock(s) I. DOI: 10.1107/S2056989024007515/ex2085Isup2.hkl

Supporting information file. DOI: 10.1107/S2056989024007515/ex2085Isup3.cml

CCDC reference: 2374683

Additional supporting information:  crystallographic information; 3D view; checkCIF report

## Figures and Tables

**Table 1:** Hydrogen-bond geometry (Å, °) *Cg* represents the centroid of the C8–C12/N2 ring.

*D*—H⋯*A*	*D*—H	H⋯*A*	*D*⋯*A*	*D*—H⋯*A*
N1—H1⋯O4^i^	0.88 (1)	2.10 (1)	2.9776 (16)	173 (1)
C19—H19*C*⋯O2^ii^	0.98	2.61	3.1785 (18)	117
C14—H14*B*⋯*Cg*^iii^	0.98	2.73	3.5955 (17)	147
C25—H25*A*⋯N1	0.99	2.65	3.3598 (17)	128
C25—H25*B*⋯O3	0.99	2.52	3.2250 (15)	128

Symmetry codes: (i) 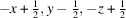; (ii) 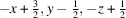; (iii) 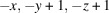.

**Table 2:** Experimental details

Crystal data	
Chemical formula	C_26_H_36_N_2_O_4_
*M*_r_	440.57
Crystal system, space group	Monoclinic, *P*2_1_/*n*
Temperature (K)	100
*a*, *b*, *c* (Å)	12.5184 (4), 9.1855 (2), 22.1339 (6)
β (°)	105.1331 (15)
*V* (Å^3^)	2456.87 (12)
*Z*	4
Radiation type	Mo *K*α
μ (mm^−1^)	0.08
Crystal size (mm)	0.42 × 0.11 × 0.06

Data collection	
Diffractometer	Bruker Kappa APEXII CCD area detector
No. of measured, independent and observed [*I* > 2σ(*I*)] reflections	23620, 5984, 4634
*R*_int_	0.031
(sin θ/λ)_max_ (Å^−1^)	0.663

Refinement	
*R*[*F*^2^ > 2σ(*F*^2^)], *wR*(*F*^2^), *S*	0.042, 0.120, 1.03
No. of reflections	5984
No. of parameters	300
No. of restraints	1
H-atom treatment	H atoms treated by a mixture of independent and constrained refinement
Δρ_max_, Δρ_min_ (e Å^−3^)	0.40, −0.24

Computer programs: *APEX2* and *SAINT* (Bruker, 2014 ▸), *SHELXS97* (Sheldrick, 2008 ▸), *SHELXL2013* (Sheldrick, 2015 ▸), *ORTEP-3 for Windows* (Farrugia, 2012 ▸) and *SHELXTL* (Sheldrick, 2008 ▸).

## supplementary crystallographic information

### {3-[(Acetyloxy)methyl]-5-{[(4,6-dimethylpyridin-2-yl)amino]methyl}-2,4,6-triethylphenyl}methyl acetate Crystal data

**Table:**

C_26_H_36_N_2_O_4_	*F*(000) = 952
*M**_r_* = 440.57	*D*_x_ = 1.191 Mg m^−^^3^
Monoclinic, *P*2_1_/*n*	Mo *K*α radiation, λ = 0.71073 Å
*a* = 12.5184 (4) Å	Cell parameters from 6078 reflections
*b* = 9.1855 (2) Å	θ = 2.4–28.3°
*c* = 22.1339 (6) Å	µ = 0.08 mm^−^^1^
β = 105.1331 (15)°	*T* = 100 K
*V* = 2456.87 (12) Å^3^	Needle, colourless
*Z* = 4	0.42 × 0.11 × 0.06 mm

### {3-[(Acetyloxy)methyl]-5-{[(4,6-dimethylpyridin-2-yl)amino]methyl}-2,4,6-triethylphenyl}methyl acetate Data collection

**Table:**

Bruker Kappa APEXII CCD area detector diffractometer	*R*_int_ = 0.031
phi and ω scans	θ_max_ = 28.1°, θ_min_ = 2.8°
23620 measured reflections	*h* = −15→16
5984 independent reflections	*k* = −12→11
4634 reflections with *I* > 2σ(*I*)	*l* = −29→29

### {3-[(Acetyloxy)methyl]-5-{[(4,6-dimethylpyridin-2-yl)amino]methyl}-2,4,6-triethylphenyl}methyl acetate Refinement

**Table:**

Refinement on *F*^2^	1 restraint
Least-squares matrix: full	Hydrogen site location: mixed
*R*[*F*^2^ > 2σ(*F*^2^)] = 0.042	H atoms treated by a mixture of independent and constrained refinement
*wR*(*F*^2^) = 0.120	*w* = 1/[σ^2^(*F*_o_^2^) + (0.0653*P*)^2^ + 0.589*P*] where *P* = (*F*_o_^2^ + 2*F*_c_^2^)/3
*S* = 1.02	(Δ/σ)_max_ < 0.001
5984 reflections	Δρ_max_ = 0.40 e Å^−^^3^
300 parameters	Δρ_min_ = −0.24 e Å^−^^3^

### {3-[(Acetyloxy)methyl]-5-{[(4,6-dimethylpyridin-2-yl)amino]methyl}-2,4,6-triethylphenyl}methyl acetate Special details

**Table:**

Geometry. All esds (except the esd in the dihedral angle between two l.s. planes) are estimated using the full covariance matrix. The cell esds are taken into account individually in the estimation of esds in distances, angles and torsion angles; correlations between esds in cell parameters are only used when they are defined by crystal symmetry. An approximate (isotropic) treatment of cell esds is used for estimating esds involving l.s. planes.

### {3-[(Acetyloxy)methyl]-5-{[(4,6-dimethylpyridin-2-yl)amino]methyl}-2,4,6-triethylphenyl}methyl acetate Fractional atomic coordinates and isotropic or equivalent isotropic displacement parameters (Å^2^)

**Table:**

	*x*	*y*	*z*	*U*_iso_*/*U*_eq_	
O1	0.57195 (8)	0.63700 (10)	0.27696 (5)	0.0206 (2)	
O2	0.75502 (8)	0.62688 (10)	0.28844 (5)	0.0251 (2)	
O3	0.11605 (8)	0.87452 (10)	0.19905 (4)	0.0185 (2)	
O4	0.13164 (10)	0.90318 (11)	0.10076 (4)	0.0284 (2)	
N1	0.27865 (10)	0.66995 (12)	0.44466 (5)	0.0184 (2)	
N2	0.22044 (10)	0.74686 (12)	0.53067 (5)	0.0187 (2)	
C1	0.35590 (11)	0.83570 (12)	0.38032 (5)	0.0133 (2)	
C2	0.45669 (10)	0.79830 (12)	0.36834 (6)	0.0136 (2)	
C3	0.47462 (10)	0.83050 (12)	0.30955 (6)	0.0135 (2)	
C4	0.39092 (11)	0.89452 (12)	0.26199 (6)	0.0129 (2)	
C5	0.28887 (10)	0.92766 (12)	0.27416 (5)	0.0122 (2)	
C6	0.27068 (10)	0.89825 (12)	0.33303 (5)	0.0125 (2)	
C7	0.33606 (11)	0.80813 (13)	0.44406 (6)	0.0166 (3)	
H7A	0.4078	0.8061	0.4762	0.020*	
H7B	0.2911	0.8883	0.4545	0.020*	
C8	0.23040 (11)	0.63821 (14)	0.49228 (6)	0.0166 (3)	
C9	0.16947 (12)	0.71630 (15)	0.57598 (6)	0.0216 (3)	
C10	0.12987 (12)	0.58025 (16)	0.58463 (6)	0.0234 (3)	
H10	0.0956	0.5637	0.6176	0.028*	
C11	0.14056 (11)	0.46587 (15)	0.54437 (6)	0.0205 (3)	
C12	0.19076 (11)	0.49616 (14)	0.49736 (6)	0.0186 (3)	
H12	0.1986	0.4224	0.4687	0.022*	
C13	0.15911 (15)	0.84358 (17)	0.61719 (7)	0.0320 (4)	
H13A	0.2330	0.8761	0.6403	0.048*	
H13B	0.1172	0.8137	0.6468	0.048*	
H13C	0.1204	0.9236	0.5912	0.048*	
C14	0.09729 (13)	0.31615 (16)	0.55260 (7)	0.0262 (3)	
H14A	0.1176	0.2486	0.5232	0.039*	
H14B	0.0165	0.3197	0.5445	0.039*	
H14C	0.1296	0.2827	0.5955	0.039*	
C15	0.54724 (11)	0.72755 (14)	0.41921 (6)	0.0186 (3)	
H15A	0.5922	0.6634	0.3996	0.022*	
H15B	0.5130	0.6664	0.4459	0.022*	
C16	0.62274 (12)	0.84108 (16)	0.46005 (7)	0.0260 (3)	
H16A	0.6584	0.9000	0.4340	0.039*	
H16B	0.6795	0.7916	0.4925	0.039*	
H16C	0.5786	0.9043	0.4798	0.039*	
C17	0.58334 (11)	0.78884 (13)	0.29743 (6)	0.0176 (3)	
H17A	0.5994	0.8517	0.2645	0.021*	
H17B	0.6442	0.7992	0.3360	0.021*	
C18	0.66651 (11)	0.56814 (14)	0.27606 (6)	0.0182 (3)	
C19	0.64560 (13)	0.41217 (15)	0.25739 (8)	0.0301 (3)	
H19A	0.6259	0.4045	0.2116	0.045*	
H19B	0.5847	0.3748	0.2732	0.045*	
H19C	0.7125	0.3550	0.2751	0.045*	
C20	0.41205 (11)	0.93293 (13)	0.19939 (6)	0.0161 (3)	
H20A	0.3421	0.9246	0.1661	0.019*	
H20B	0.4656	0.8629	0.1899	0.019*	
C21	0.45783 (12)	1.08797 (14)	0.19947 (7)	0.0217 (3)	
H21A	0.4083	1.1567	0.2125	0.033*	
H21B	0.4629	1.1129	0.1573	0.033*	
H21C	0.5315	1.0932	0.2287	0.033*	
C22	0.19501 (11)	0.99175 (13)	0.22362 (6)	0.0154 (3)	
H22A	0.1582	1.0705	0.2412	0.018*	
H22B	0.2238	1.0332	0.1897	0.018*	
C23	0.09262 (11)	0.84169 (14)	0.13812 (6)	0.0171 (3)	
C24	0.01268 (13)	0.71748 (16)	0.12222 (6)	0.0248 (3)	
H24A	−0.0613	0.7550	0.1019	0.037*	
H24B	0.0107	0.6656	0.1606	0.037*	
H24C	0.0363	0.6506	0.0937	0.037*	
C25	0.16236 (11)	0.93941 (13)	0.34683 (6)	0.0155 (2)	
H25A	0.1467	0.8708	0.3780	0.019*	
H25B	0.1016	0.9312	0.3080	0.019*	
C26	0.16604 (12)	1.09505 (14)	0.37219 (7)	0.0229 (3)	
H26A	0.2244	1.1025	0.4114	0.034*	
H26B	0.0945	1.1191	0.3800	0.034*	
H26C	0.1815	1.1631	0.3415	0.034*	
H1	0.3047 (13)	0.5952 (13)	0.4283 (7)	0.022 (4)*	

### {3-[(Acetyloxy)methyl]-5-{[(4,6-dimethylpyridin-2-yl)amino]methyl}-2,4,6-triethylphenyl}methyl acetate Atomic displacement parameters (Å^2^)

**Table:**

	*U*^11^	*U*^22^	*U*^33^	*U*^12^	*U*^13^	*U*^23^
O1	0.0143 (5)	0.0159 (5)	0.0321 (5)	0.0015 (3)	0.0069 (4)	−0.0065 (4)
O2	0.0159 (5)	0.0221 (5)	0.0394 (6)	0.0014 (4)	0.0107 (4)	0.0042 (4)
O3	0.0164 (5)	0.0236 (5)	0.0143 (4)	−0.0058 (4)	0.0019 (3)	0.0013 (3)
O4	0.0416 (7)	0.0271 (5)	0.0184 (5)	−0.0097 (5)	0.0111 (4)	0.0003 (4)
N1	0.0270 (7)	0.0129 (5)	0.0186 (5)	−0.0006 (4)	0.0117 (5)	−0.0002 (4)
N2	0.0195 (6)	0.0203 (5)	0.0172 (5)	0.0008 (4)	0.0065 (4)	−0.0007 (4)
C1	0.0170 (6)	0.0103 (5)	0.0127 (5)	−0.0019 (4)	0.0038 (4)	−0.0007 (4)
C2	0.0149 (6)	0.0094 (5)	0.0152 (6)	−0.0004 (4)	0.0014 (5)	−0.0012 (4)
C3	0.0131 (6)	0.0096 (5)	0.0181 (6)	−0.0014 (4)	0.0046 (5)	−0.0025 (4)
C4	0.0159 (6)	0.0087 (5)	0.0150 (6)	−0.0016 (4)	0.0052 (5)	−0.0013 (4)
C5	0.0129 (6)	0.0093 (5)	0.0138 (5)	−0.0008 (4)	0.0024 (4)	−0.0013 (4)
C6	0.0138 (6)	0.0093 (5)	0.0149 (6)	−0.0009 (4)	0.0047 (4)	−0.0016 (4)
C7	0.0219 (7)	0.0151 (6)	0.0130 (6)	−0.0013 (5)	0.0046 (5)	0.0006 (4)
C8	0.0163 (7)	0.0191 (6)	0.0141 (6)	0.0024 (5)	0.0037 (5)	0.0032 (5)
C9	0.0211 (7)	0.0271 (7)	0.0184 (6)	0.0025 (5)	0.0085 (5)	−0.0010 (5)
C10	0.0239 (8)	0.0300 (7)	0.0193 (6)	−0.0005 (6)	0.0110 (5)	0.0028 (5)
C11	0.0168 (7)	0.0226 (7)	0.0218 (6)	0.0015 (5)	0.0046 (5)	0.0057 (5)
C12	0.0202 (7)	0.0181 (6)	0.0183 (6)	0.0024 (5)	0.0065 (5)	0.0022 (5)
C13	0.0421 (10)	0.0322 (8)	0.0286 (8)	−0.0012 (7)	0.0214 (7)	−0.0069 (6)
C14	0.0265 (8)	0.0247 (7)	0.0291 (7)	−0.0021 (6)	0.0107 (6)	0.0070 (6)
C15	0.0179 (7)	0.0164 (6)	0.0188 (6)	0.0042 (5)	−0.0002 (5)	0.0010 (5)
C16	0.0218 (8)	0.0269 (7)	0.0234 (7)	0.0033 (6)	−0.0046 (5)	−0.0039 (6)
C17	0.0145 (6)	0.0149 (6)	0.0241 (6)	−0.0002 (5)	0.0061 (5)	−0.0034 (5)
C18	0.0164 (7)	0.0194 (6)	0.0207 (6)	0.0044 (5)	0.0079 (5)	0.0036 (5)
C19	0.0254 (8)	0.0191 (7)	0.0486 (9)	0.0040 (6)	0.0144 (7)	−0.0057 (6)
C20	0.0188 (7)	0.0154 (6)	0.0162 (6)	0.0001 (5)	0.0081 (5)	−0.0001 (5)
C21	0.0279 (8)	0.0163 (6)	0.0257 (7)	−0.0013 (5)	0.0157 (6)	0.0026 (5)
C22	0.0155 (6)	0.0141 (6)	0.0157 (6)	0.0001 (5)	0.0024 (5)	0.0011 (4)
C23	0.0168 (7)	0.0174 (6)	0.0156 (6)	0.0028 (5)	0.0018 (5)	0.0027 (5)
C24	0.0233 (8)	0.0270 (7)	0.0201 (6)	−0.0070 (6)	−0.0015 (5)	0.0009 (5)
C25	0.0127 (6)	0.0173 (6)	0.0173 (6)	0.0004 (5)	0.0053 (5)	−0.0015 (5)
C26	0.0248 (8)	0.0190 (6)	0.0260 (7)	0.0052 (5)	0.0085 (6)	−0.0033 (5)

### {3-[(Acetyloxy)methyl]-5-{[(4,6-dimethylpyridin-2-yl)amino]methyl}-2,4,6-triethylphenyl}methyl acetate Geometric parameters (Å, º)

**Table:**

O1—C18	1.3469 (16)	C13—H13C	0.9800
O1—C17	1.4619 (15)	C14—H14A	0.9800
O2—C18	1.1980 (17)	C14—H14B	0.9800
O3—C23	1.3377 (15)	C14—H14C	0.9800
O3—C22	1.4669 (15)	C15—C16	1.5336 (19)
O4—C23	1.2051 (16)	C15—H15A	0.9900
N1—C8	1.3761 (16)	C15—H15B	0.9900
N1—C7	1.4603 (16)	C16—H16A	0.9800
N1—H1	0.878 (9)	C16—H16B	0.9800
N2—C8	1.3375 (17)	C16—H16C	0.9800
N2—C9	1.3511 (17)	C17—H17A	0.9900
C1—C2	1.3982 (18)	C17—H17B	0.9900
C1—C6	1.4073 (17)	C18—C19	1.4952 (19)
C1—C7	1.5164 (16)	C19—H19A	0.9800
C2—C3	1.4083 (17)	C19—H19B	0.9800
C2—C15	1.5194 (17)	C19—H19C	0.9800
C3—C4	1.4057 (17)	C20—C21	1.5349 (17)
C3—C17	1.5038 (18)	C20—H20A	0.9900
C4—C5	1.4060 (17)	C20—H20B	0.9900
C4—C20	1.5185 (16)	C21—H21A	0.9800
C5—C6	1.4063 (16)	C21—H21B	0.9800
C5—C22	1.5140 (16)	C21—H21C	0.9800
C6—C25	1.5137 (17)	C22—H22A	0.9900
C7—H7A	0.9900	C22—H22B	0.9900
C7—H7B	0.9900	C23—C24	1.4977 (19)
C8—C12	1.4108 (18)	C24—H24A	0.9800
C9—C10	1.376 (2)	C24—H24B	0.9800
C9—C13	1.5092 (19)	C24—H24C	0.9800
C10—C11	1.406 (2)	C25—C26	1.5322 (17)
C10—H10	0.9500	C25—H25A	0.9900
C11—C12	1.3770 (18)	C25—H25B	0.9900
C11—C14	1.5064 (19)	C26—H26A	0.9800
C12—H12	0.9500	C26—H26B	0.9800
C13—H13A	0.9800	C26—H26C	0.9800
C13—H13B	0.9800		
			
C18—O1—C17	115.95 (10)	H15A—C15—H15B	107.9
C23—O3—C22	119.24 (10)	C15—C16—H16A	109.5
C8—N1—C7	120.44 (10)	C15—C16—H16B	109.5
C8—N1—H1	115.8 (11)	H16A—C16—H16B	109.5
C7—N1—H1	116.1 (11)	C15—C16—H16C	109.5
C8—N2—C9	117.18 (12)	H16A—C16—H16C	109.5
C2—C1—C6	120.38 (11)	H16B—C16—H16C	109.5
C2—C1—C7	120.69 (11)	O1—C17—C3	106.17 (10)
C6—C1—C7	118.92 (11)	O1—C17—H17A	110.5
C1—C2—C3	119.53 (11)	C3—C17—H17A	110.5
C1—C2—C15	120.01 (11)	O1—C17—H17B	110.5
C3—C2—C15	120.43 (11)	C3—C17—H17B	110.5
C4—C3—C2	120.82 (11)	H17A—C17—H17B	108.7
C4—C3—C17	120.35 (11)	O2—C18—O1	123.31 (12)
C2—C3—C17	118.78 (11)	O2—C18—C19	125.41 (13)
C3—C4—C5	118.95 (11)	O1—C18—C19	111.28 (12)
C3—C4—C20	120.47 (11)	C18—C19—H19A	109.5
C5—C4—C20	120.53 (11)	C18—C19—H19B	109.5
C4—C5—C6	120.72 (11)	H19A—C19—H19B	109.5
C4—C5—C22	120.75 (10)	C18—C19—H19C	109.5
C6—C5—C22	118.50 (11)	H19A—C19—H19C	109.5
C5—C6—C1	119.53 (11)	H19B—C19—H19C	109.5
C5—C6—C25	120.62 (11)	C4—C20—C21	111.62 (10)
C1—C6—C25	119.80 (11)	C4—C20—H20A	109.3
N1—C7—C1	110.74 (10)	C21—C20—H20A	109.3
N1—C7—H7A	109.5	C4—C20—H20B	109.3
C1—C7—H7A	109.5	C21—C20—H20B	109.3
N1—C7—H7B	109.5	H20A—C20—H20B	108.0
C1—C7—H7B	109.5	C20—C21—H21A	109.5
H7A—C7—H7B	108.1	C20—C21—H21B	109.5
N2—C8—N1	117.44 (11)	H21A—C21—H21B	109.5
N2—C8—C12	123.12 (12)	C20—C21—H21C	109.5
N1—C8—C12	119.40 (11)	H21A—C21—H21C	109.5
N2—C9—C10	123.30 (12)	H21B—C21—H21C	109.5
N2—C9—C13	114.79 (12)	O3—C22—C5	107.84 (9)
C10—C9—C13	121.90 (13)	O3—C22—H22A	110.1
C9—C10—C11	119.53 (12)	C5—C22—H22A	110.1
C9—C10—H10	120.2	O3—C22—H22B	110.1
C11—C10—H10	120.2	C5—C22—H22B	110.1
C12—C11—C10	117.74 (13)	H22A—C22—H22B	108.5
C12—C11—C14	121.68 (13)	O4—C23—O3	124.38 (12)
C10—C11—C14	120.58 (12)	O4—C23—C24	124.16 (12)
C11—C12—C8	119.11 (12)	O3—C23—C24	111.45 (11)
C11—C12—H12	120.4	C23—C24—H24A	109.5
C8—C12—H12	120.4	C23—C24—H24B	109.5
C9—C13—H13A	109.5	H24A—C24—H24B	109.5
C9—C13—H13B	109.5	C23—C24—H24C	109.5
H13A—C13—H13B	109.5	H24A—C24—H24C	109.5
C9—C13—H13C	109.5	H24B—C24—H24C	109.5
H13A—C13—H13C	109.5	C6—C25—C26	111.35 (11)
H13B—C13—H13C	109.5	C6—C25—H25A	109.4
C11—C14—H14A	109.5	C26—C25—H25A	109.4
C11—C14—H14B	109.5	C6—C25—H25B	109.4
H14A—C14—H14B	109.5	C26—C25—H25B	109.4
C11—C14—H14C	109.5	H25A—C25—H25B	108.0
H14A—C14—H14C	109.5	C25—C26—H26A	109.5
H14B—C14—H14C	109.5	C25—C26—H26B	109.5
C2—C15—C16	111.82 (10)	H26A—C26—H26B	109.5
C2—C15—H15A	109.3	C25—C26—H26C	109.5
C16—C15—H15A	109.3	H26A—C26—H26C	109.5
C2—C15—H15B	109.3	H26B—C26—H26C	109.5
C16—C15—H15B	109.3		
			
C6—C1—C2—C3	3.27 (17)	C7—N1—C8—N2	11.94 (18)
C7—C1—C2—C3	−177.42 (11)	C7—N1—C8—C12	−170.12 (12)
C6—C1—C2—C15	−178.56 (11)	C8—N2—C9—C10	1.1 (2)
C7—C1—C2—C15	0.75 (17)	C8—N2—C9—C13	−179.11 (13)
C1—C2—C3—C4	−2.52 (17)	N2—C9—C10—C11	−0.9 (2)
C15—C2—C3—C4	179.31 (11)	C13—C9—C10—C11	179.24 (14)
C1—C2—C3—C17	−179.72 (11)	C9—C10—C11—C12	−0.1 (2)
C15—C2—C3—C17	2.11 (17)	C9—C10—C11—C14	−179.63 (13)
C2—C3—C4—C5	0.69 (17)	C10—C11—C12—C8	1.0 (2)
C17—C3—C4—C5	177.86 (10)	C14—C11—C12—C8	−179.52 (12)
C2—C3—C4—C20	178.20 (10)	N2—C8—C12—C11	−0.9 (2)
C17—C3—C4—C20	−4.63 (17)	N1—C8—C12—C11	−178.70 (12)
C3—C4—C5—C6	0.39 (17)	C1—C2—C15—C16	−88.79 (15)
C20—C4—C5—C6	−177.12 (10)	C3—C2—C15—C16	89.37 (14)
C3—C4—C5—C22	−177.83 (10)	C18—O1—C17—C3	−166.61 (11)
C20—C4—C5—C22	4.67 (17)	C4—C3—C17—O1	−92.28 (13)
C4—C5—C6—C1	0.36 (17)	C2—C3—C17—O1	84.94 (13)
C22—C5—C6—C1	178.61 (10)	C17—O1—C18—O2	−3.15 (18)
C4—C5—C6—C25	177.60 (11)	C17—O1—C18—C19	177.29 (11)
C22—C5—C6—C25	−4.15 (16)	C3—C4—C20—C21	−89.67 (14)
C2—C1—C6—C5	−2.21 (17)	C5—C4—C20—C21	87.81 (14)
C7—C1—C6—C5	178.47 (10)	C23—O3—C22—C5	−121.28 (12)
C2—C1—C6—C25	−179.47 (11)	C4—C5—C22—O3	102.10 (12)
C7—C1—C6—C25	1.21 (16)	C6—C5—C22—O3	−76.15 (13)
C8—N1—C7—C1	−165.33 (11)	C22—O3—C23—O4	−0.62 (19)
C2—C1—C7—N1	−95.56 (14)	C22—O3—C23—C24	178.70 (11)
C6—C1—C7—N1	83.76 (14)	C5—C6—C25—C26	−88.44 (13)
C9—N2—C8—N1	177.71 (12)	C1—C6—C25—C26	88.80 (14)
C9—N2—C8—C12	−0.15 (19)		

### {3-[(Acetyloxy)methyl]-5-{[(4,6-dimethylpyridin-2-yl)amino]methyl}-2,4,6-triethylphenyl}methyl acetate Hydrogen-bond geometry (Å, º)

*Cg* represents the centroid of the C8–C12/N2 ring.

**Table:**

*D*—H···*A*	*D*—H	H···*A*	*D*···*A*	*D*—H···*A*
N1—H1···O4^i^	0.88 (1)	2.10 (1)	2.9776 (16)	173 (1)
C19—H19*C*···O2^ii^	0.98	2.61	3.1785 (18)	117
C14—H14*B*···*Cg*^iii^	0.98	2.73	3.5955 (17)	147
C25—H25*A*···N1	0.99	2.65	3.3598 (17)	128
C25—H25*B*···O3	0.99	2.52	3.2250 (15)	128

Symmetry codes: (i) −*x*+1/2, *y*−1/2, −*z*+1/2; (ii) −*x*+3/2, *y*−1/2, −*z*+1/2; (iii) −*x*, −*y*+1, −*z*+1.
